# 4-Chloro­benzothio­amide

**DOI:** 10.1107/S1600536809014640

**Published:** 2009-04-25

**Authors:** Mahmood-ul-Hassan Khan, Shahid Hameed, Tashfeen Akhtar, Jason D. Masuda

**Affiliations:** a Department of Chemistry, Quaid-i-Azam University, Islamabad 45320, Pakistan; b Department of Chemistry, Saint Mary’s University, Halifax, Nova Scotia, Canada B3H 3C3

## Abstract

In the title compound, C_7_H_6_ClNS, the dihedral angle between the aromatic ring and the thio­amide fragment is 28.1 (2)°. The structure features a π-stacking inter­action between the aromatic rings with a slight offset of the rings, giving a centroid–centroid separation of 3.7942 (2) Å. There are inter­molecular hydrogen-bonding inter­actions between the amino group and the S atoms.

## Related literature

For the uses of thio­amides, see: Akhtar *et al.* (2006 ▶, 2007 ▶, 2008 ▶); Jagodzinski (2003 ▶); Lebana *et al.* (2008 ▶). For the biological activity of thio­amides, see: Wei *et al.* (2006 ▶). For the synthesis of thio­amides, see: Bauer & Kuhlein (1985 ▶); Cava & Levinson (1985 ▶); Manaka & Sato (2005 ▶). For a comparable structure, see: Jian *et al.* (2006 ▶).
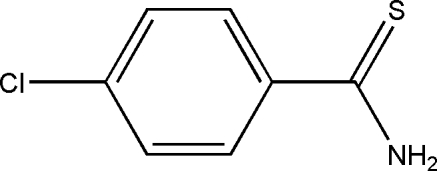


## Experimental

### 

#### Crystal data

C_7_H_6_ClNS
*M* *_r_* = 171.64
Monoclinic, 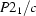

*a* = 8.1592 (4) Å
*b* = 9.0934 (5) Å
*c* = 10.8915 (6) Å
β = 100.113 (10°
*V* = 795.54 (7) Å^3^
*Z* = 4
Mo *K*α radiation
μ = 0.66 mm^−1^
*T* = 296 K
0.40 × 0.36 × 0.18 mm

#### Data collection

Bruker APEXII CCD diffractometer
Absorption correction: multi-scan (*SADABS*; Bruker, 2008 ▶) *T*_min_ = 0.778, *T*_max_ = 0.889
6337 measured reflections
1901 independent reflections
1667 reflections with *I* > 2σ(*I*)
*R*_int_ = 0.017

#### Refinement

*R*[*F* ^2^ > 2σ(*F* ^2^)] = 0.037
*wR*(*F* ^2^) = 0.105
*S* = 1.06
1901 reflections
91 parameters
H-atom parameters constrained
Δρ_max_ = 0.33 e Å^−3^
Δρ_min_ = −0.38 e Å^−3^

### 

Data collection: *APEX2* (Bruker, 2008 ▶); cell refinement: *SAINT* (Bruker, 2008 ▶); data reduction: *SAINT*; program(s) used to solve structure: *SHELXS97* (Sheldrick, 2008 ▶); program(s) used to refine structure: *SHELXL97* (Sheldrick, 2008 ▶); molecular graphics: *ORTEP-3 for Windows* (Farrugia, 1997 ▶); software used to prepare material for publication: *SHELXTL* (Sheldrick, 2008 ▶).

## Supplementary Material

Crystal structure: contains datablocks I, global. DOI: 10.1107/S1600536809014640/bt2933sup1.cif

Structure factors: contains datablocks I. DOI: 10.1107/S1600536809014640/bt2933Isup2.hkl

Additional supplementary materials:  crystallographic information; 3D view; checkCIF report

## Figures and Tables

**Table 1:** Hydrogen-bond geometry (Å, °)

*D*—H⋯*A*	*D*—H	H⋯*A*	*D*⋯*A*	*D*—H⋯*A*
N1—H1*A*⋯S1^i^	0.86	2.64	3.3769 (15)	145
N1—H1*B*⋯S1^ii^	0.86	2.63	3.4527 (15)	160

Symmetry codes: (i) 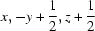; (ii) 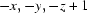.

**Fig. 1.**
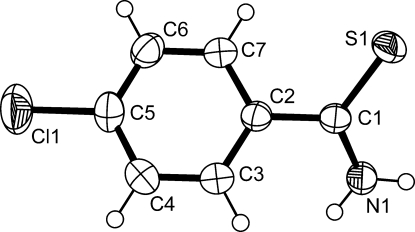
Molecular structure of 4-chlorobenzothioamide showing displacement ellipsoids at the 50% probability level (for non-H atoms).

**Fig. 2.**
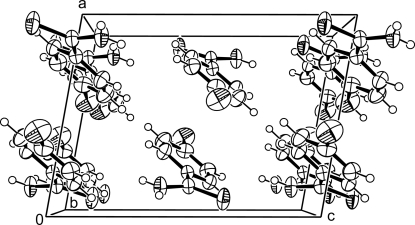
Packing diagram of 4-chlorobenzothioamide as viewed down the b axis. Displacement ellipsoids are shown at the 50% probability level (for non-H atoms).

## supplementary crystallographic information

### Comment

Thioamides are important precursors/intermediates in the synthesis of various heterocycles (Jagodzinski *et al.*, 2003). Besides being used as synthetic intermediates, they exhibit numerous biological activities (Wei *et al.*, 2006). In addition, thioamides have found use as important ligands in coordination chemistry (Lebana *et al.*, 2008). Several methods for their synthesis have been published involving the uses of Lawesson's regent (Cava *et al.*, 1985) and phosphorus pentasulphide (Bauer *et al.*, 1985). The title compound, 4-chlorobenzothioamide was synthesized in continuation of our previous work on the synthesis and biological screenings of five membered heterocycles (Akhtar *et al.*, 2006, 2007, 2008). In this article the crystal structure of 4-chlorobenzothioamide is being reported. The title compound was synthesized by treating 4-chlorobenzonitrile with 70% sodium hydrogen sulfide hydrate and magnesium chloride hexahydrate in dimethylformamide (Manaka & Sato, 2005) as an intermediate for the synthesis of thiazoles.

The hydrogen bonding interactions between the nitrogen and sulfur atoms are in the range of those seen in *p*-trifluoromethylbenzothioamide where the corresponding interactions are between 3.3735Å and 3.5133Å (Jian *et al.*, 2006).

### Experimental

4-Chlorobenzonitrile (14.5 mmol) was added to a slurry of sodium hydrogen sulfide hydrate (70%, 29 mmol) and magnesium chloride hexahydrate (14.5 mmol) in DMF (40 mL) and the mixture stirred at room temperature for 2 h. The resulting green slurry was poured into water (100 mL) and the precipitated solid collected by filtration. The product obtained was resuspended in 1 N HCl (50 ml), stirred for another 30 min, filtered and washed with excess of water. The recrystallization of the residue from chloroform afforded the crystals of the title compound suitable for X-ray analysis.

### Refinement

The hydrogen atoms were placed in geometrically idealized positions of 0.93Å (aromatic C—H) and 0.86Å (amide N—H) and constrained to ride on the parent atom with *U*_iso_(H) = 1.2 U_eq_(C,N).

### Crystal data

**Table:**

C_7_H_6_ClNS	*F*(000) = 352
*M**_r_* = 171.64	*D*_x_ = 1.433 Mg m^−^^3^
Monoclinic, *P*2_1_/*c*	Mo *K*α radiation, λ = 0.71073 Å
Hall symbol: -P 2ybc	Cell parameters from 3894 reflections
*a* = 8.1592 (4) Å	θ = 2.5–28.5°
*b* = 9.0934 (5) Å	µ = 0.66 mm^−^^1^
*c* = 10.8915 (6) Å	*T* = 296 K
β = 100.113 (1)°	Block, yellow
*V* = 795.54 (7) Å^3^	0.40 × 0.36 × 0.18 mm
*Z* = 4	

### Data collection

**Table:**

Bruker APEXII CCD diffractometer	1901 independent reflections
Radiation source: fine-focus sealed tube	1667 reflections with *I* > 2σ(*I*)
graphite	*R*_int_ = 0.017
φ and ω scans	θ_max_ = 28.5°, θ_min_ = 2.9°
Absorption correction: multi-scan (*SADABS*; Bruker, 2008)	*h* = −10→10
*T*_min_ = 0.778, *T*_max_ = 0.889	*k* = −12→12
6337 measured reflections	*l* = −9→14

### Refinement

**Table:**

Refinement on *F*^2^	Primary atom site location: structure-invariant direct methods
Least-squares matrix: full	Secondary atom site location: difference Fourier map
*R*[*F*^2^ > 2σ(*F*^2^)] = 0.037	Hydrogen site location: inferred from neighbouring sites
*wR*(*F*^2^) = 0.105	H-atom parameters constrained
*S* = 1.06	*w* = 1/[σ^2^(*F*_o_^2^) + (0.0539*P*)^2^ + 0.2817*P*] where *P* = (*F*_o_^2^ + 2*F*_c_^2^)/3
1901 reflections	(Δ/σ)_max_ < 0.001
91 parameters	Δρ_max_ = 0.33 e Å^−^^3^
0 restraints	Δρ_min_ = −0.38 e Å^−^^3^

### Special details

**Table:**

Geometry. All e.s.d.'s (except the e.s.d. in the dihedral angle between two l.s. planes) are estimated using the full covariance matrix. The cell e.s.d.'s are taken into account individually in the estimation of e.s.d.'s in distances, angles and torsion angles; correlations between e.s.d.'s in cell parameters are only used when they are defined by crystal symmetry. An approximate (isotropic) treatment of cell e.s.d.'s is used for estimating e.s.d.'s involving l.s. planes.
Refinement. Refinement of *F*^2^ against ALL reflections. The weighted *R*-factor *wR* and goodness of fit *S* are based on *F*^2^, conventional *R*-factors *R* are based on *F*, with *F* set to zero for negative *F*^2^. The threshold expression of *F*^2^ > σ(*F*^2^) is used only for calculating *R*-factors(gt) *etc*. and is not relevant to the choice of reflections for refinement. *R*-factors based on *F*^2^ are statistically about twice as large as those based on *F*, and *R*- factors based on ALL data will be even larger.

### Fractional atomic coordinates and isotropic or equivalent isotropic displacement parameters (Å^2^)

**Table:**

	*x*	*y*	*z*	*U*_iso_*/*U*_eq_	
S1	0.08443 (7)	0.15260 (5)	0.36191 (4)	0.05425 (18)	
Cl1	0.41127 (8)	0.84764 (5)	0.55354 (7)	0.0767 (2)	
N1	0.1431 (2)	0.16063 (15)	0.60516 (12)	0.0479 (4)	
H1A	0.1776	0.2025	0.6758	0.058*	
H1B	0.1046	0.0724	0.6028	0.058*	
C1	0.14913 (18)	0.23175 (17)	0.50045 (13)	0.0367 (3)	
C2	0.21721 (18)	0.38334 (16)	0.51285 (13)	0.0346 (3)	
C7	0.16710 (19)	0.48716 (18)	0.41971 (14)	0.0402 (3)	
H7A	0.0925	0.4603	0.3485	0.048*	
C5	0.3367 (2)	0.66844 (17)	0.53788 (18)	0.0466 (4)	
C6	0.2270 (2)	0.62963 (18)	0.43192 (17)	0.0462 (4)	
H6A	0.1937	0.6984	0.3693	0.055*	
C3	0.3294 (2)	0.42569 (19)	0.61821 (15)	0.0448 (4)	
H3A	0.3647	0.3573	0.6808	0.054*	
C4	0.3893 (2)	0.5685 (2)	0.63118 (17)	0.0515 (4)	
H4A	0.4641	0.5963	0.7020	0.062*	

### Atomic displacement parameters (Å^2^)

**Table:**

	*U*^11^	*U*^22^	*U*^33^	*U*^12^	*U*^13^	*U*^23^
S1	0.0851 (4)	0.0495 (3)	0.0281 (2)	−0.0239 (2)	0.0096 (2)	−0.00374 (15)
Cl1	0.0841 (4)	0.0399 (3)	0.1008 (5)	−0.0176 (2)	0.0012 (3)	−0.0009 (2)
N1	0.0790 (10)	0.0363 (7)	0.0299 (7)	−0.0104 (6)	0.0134 (6)	−0.0014 (5)
C1	0.0449 (7)	0.0369 (7)	0.0291 (7)	−0.0020 (6)	0.0091 (6)	−0.0005 (5)
C2	0.0393 (7)	0.0345 (7)	0.0307 (7)	−0.0002 (5)	0.0085 (5)	−0.0002 (5)
C7	0.0438 (7)	0.0415 (8)	0.0341 (8)	0.0001 (6)	0.0030 (6)	0.0029 (6)
C5	0.0457 (8)	0.0337 (7)	0.0604 (11)	−0.0048 (6)	0.0095 (7)	−0.0024 (7)
C6	0.0489 (8)	0.0383 (8)	0.0504 (10)	0.0020 (7)	0.0056 (7)	0.0095 (7)
C3	0.0520 (9)	0.0422 (8)	0.0374 (8)	−0.0029 (7)	−0.0002 (6)	0.0040 (6)
C4	0.0539 (9)	0.0483 (9)	0.0477 (10)	−0.0087 (7)	−0.0036 (7)	−0.0042 (7)

### Geometric parameters (Å, °)

**Table:**

S1—C1	1.6714 (15)	C7—C6	1.383 (2)
Cl1—C5	1.7374 (16)	C7—H7A	0.9300
N1—C1	1.3195 (19)	C5—C4	1.375 (3)
N1—H1A	0.8600	C5—C6	1.376 (3)
N1—H1B	0.8600	C6—H6A	0.9300
C1—C2	1.483 (2)	C3—C4	1.386 (2)
C2—C3	1.391 (2)	C3—H3A	0.9300
C2—C7	1.393 (2)	C4—H4A	0.9300
			
C1—N1—H1A	120.0	C4—C5—C6	121.55 (15)
C1—N1—H1B	120.0	C4—C5—Cl1	119.26 (14)
H1A—N1—H1B	120.0	C6—C5—Cl1	119.19 (14)
N1—C1—C2	116.55 (13)	C5—C6—C7	119.19 (15)
N1—C1—S1	121.02 (12)	C5—C6—H6A	120.4
C2—C1—S1	122.42 (11)	C7—C6—H6A	120.4
C3—C2—C7	118.65 (14)	C4—C3—C2	120.85 (15)
C3—C2—C1	120.94 (14)	C4—C3—H3A	119.6
C7—C2—C1	120.40 (13)	C2—C3—H3A	119.6
C6—C7—C2	120.74 (15)	C5—C4—C3	119.02 (15)
C6—C7—H7A	119.6	C5—C4—H4A	120.5
C2—C7—H7A	119.6	C3—C4—H4A	120.5
			
S1—C1—C2—C7	28.1 (2)		

### Hydrogen-bond geometry (Å, °)

**Table:**

*D*—H···*A*	*D*—H	H···*A*	*D*···*A*	*D*—H···*A*
N1—H1A···S1^i^	0.86	2.64	3.3769 (15)	145
N1—H1B···S1^ii^	0.86	2.63	3.4527 (15)	160

Symmetry codes: (i) *x*, −*y*+1/2, *z*+1/2; (ii) −*x*, −*y*, −*z*+1.
